# Pharmacokinetic Interaction Between Prasugrel and Ritonavir in Healthy Volunteers

**DOI:** 10.1111/j.1742-7843.2012.00932.x

**Published:** 2012-10-05

**Authors:** Virginie Ancrenaz, Julien Déglon, Caroline Samer, Christian Staub, Pierre Dayer, Youssef Daali, Jules Desmeules

**Affiliations:** 1 Clinical Pharmacology and Toxicology Service, Geneva University Hospitals and Faculty of MedicineGeneva, Switzerland; 2 Unit of Toxicology, University Center of Legal MedicineGeneva, Switzerland; 3 Swiss Center of Applied Human Toxicology, Geneva UniversityGeneva, Switzerland

## Abstract

The new anti-aggregating agent prasugrel is bioactivated by cytochromes P450 (CYP) 3A and 2B6. Ritonavir is a potent CYP3A inhibitor and was shown *in vitro* as a CYP2B6 inhibitor. The aim of this open-label cross-over study was to assess the effect of ritonavir on prasugrel active metabolite (prasugrel AM) pharmacokinetics in healthy volunteers. Ten healthy male volunteers received 10 mg prasugrel. After at least a week washout, they received 100 mg ritonavir, followed by 10 mg prasugrel 2 hr later. We used dried blood spot sampling method to monitor prasugrel AM pharmacokinetics (*C*_max_, *t*_1/2_, *t*_max_, AUC_0–6 hr_) at 0, 0.25, 0.5, 1, 1.5, 2, 4 and 6 hr after prasugrel administration. A ‘cocktail’ approach was used to measure CYP2B6, 2C9, 2C19 and 3A activities. In the presence of ritonavir, prasugrel AM *C*_max_ and AUC were decreased by 45% (mean ratio: 0.55, CI 90%: 0.40–0.7, *p = 0.007*) and 38% (mean ratio: 0.62, CI 90%: 0.54–0.7, *p = 0.005*), respectively, while *t*_1/2_ and *t*_max_ were not affected. Midazolam metabolic ratio (MR) dramatically decreased in presence of ritonavir (6.7 ± 2.6 *versus* 0.13 ± 0.07) reflecting an almost complete inhibition of CYP3A4, whereas omeprazole, flurbiprofen and bupropion MR were not affected. These data demonstrate that ritonavir is able to block prasugrel CYP3A4 bioactivation. This CYP-mediated drug–drug interaction might lead to a significant reduction of prasugrel efficacy in HIV-infected patients with acute coronary syndrome.

## Introduction

Prasugrel is a recently commercialized thienopyridine antiplatelet agent used to prevent atherothrombotic events in patients with acute coronary syndrome who are undergoing percutaneous coronary intervention [1].

**Fig. 1:**
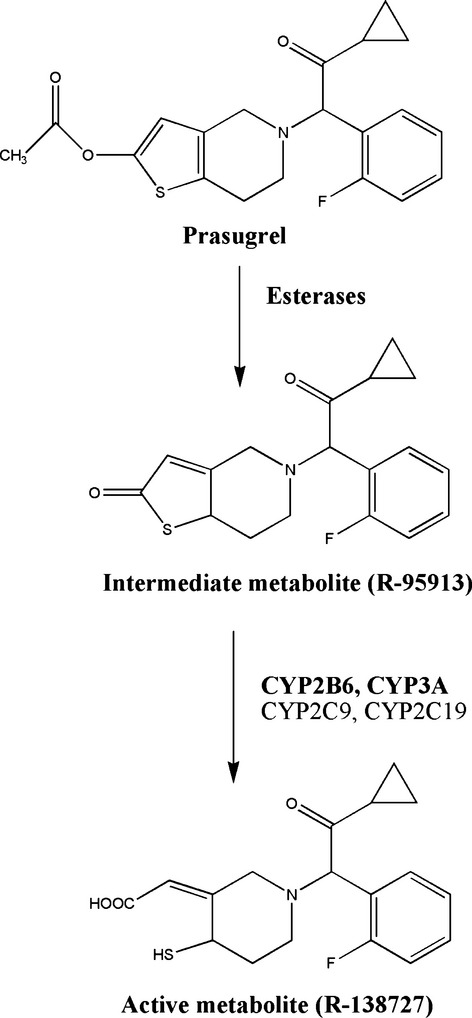
Metabolic pathways of the anti-aggregating agent prasugrel [8].

The active metabolite of prasugrel (prasugrel AM) is an irreversible inhibitor of ADP-P2Y12 platelet receptors, explaining the anti-aggregating effect of prasugrel. This pro-drug is rapidly hydrolysed by carboxylesterases into a thiolactone intermediate metabolite that is transformed by cytochromes P450 (CYP) into its pharmacologically active metabolite (fig. 1). CYP3A and CYP2B6 are primarily responsible for this transformation while CYP2C19 and CYP2C9 are involved to a lesser extent [2]. CYP3A and 2B6 represent, respectively, 40% and 5% of the total cytochromes in the human liver and they are known to be involved in the oxidative metabolism of a wide range of drugs in clinical use [3,4]. Consequently, *in vivo* interactions between prasugrel and other drugs affecting these CYPs are possible. The effects of some CYP inhibitors or inducers on prasugrel bioactivation have been assessed previously. For example, there was no change in prasugrel AM formation or the inhibition of platelet aggregation when prasugrel was administered with ketoconazole (a CYP3A inhibitor), atorvastatin (a CYP3A substrate) or rifampicin (a CYP3A inducer) [5–7]. These studies suggest that the production of prasugrel AM is not altered by induction or inhibition of CYP3A. However, an *in vitro* study showed that prasugrel was mainly metabolized by CYP3A and CYP2B6 and simultaneous inhibition of these two pathways significantly decreased prasugrel bioactivation. Indeed, ritonavir was a potent inhibitor of prasugrel bioactivation in human liver microsomes, raising the possibility of a pharmacokinetic drug–drug interaction between ritonavir and prasugrel *in vivo* [8]. Such an interaction could reduce the efficacy of prasugrel. *In vivo* experiments are lacking to confirm these *in vitro* data in human beings.

Ritonavir is an antiretroviral agent widely used as a pharmacokinetic booster in patients with HIV infection to increase the plasma concentrations of other antiretroviral drugs. In HIV patients with acute coronary syndrome, these two drugs could be largely prescribed concomitantly. Indeed, ritonavir is known to be a potent inhibitor of CYP3A and CYP2B6 and is a weaker inhibitor of CYP2C9 [8–12].

While blood volume is seldom a major limitation of clinical trials, the invasiveness of phlebotomy certainly is. Compared with classic venipuncture, dried blood spots (DBS) sampling is less invasive and can be easily performed. Additionally, when shipping and storing samples, DBS sampling eliminates the need for dry ice, plasma separation and addition of anticoagulants that are required to maintain the viability of traditional blood samples. Regarding these benefits, DBS sampling has grown in popularity in the clinical and the pharmaceutical communities over the past decade. Recent publications demonstrated that DBS is a viable approach for the quantitative measurement of drugs and metabolites both in human beings and animals [13–15].

In our study, we assessed the potential pharmacokinetic interaction between ritonavir and prasugrel in healthy volunteers. The activities of the CYPs involved in prasugrel metabolism were also evaluated using a cocktail approach.

## Material and Methods

### Volunteers

Inclusion criteria required that volunteers be healthy males between 18 and 60 years of age, have a body mass index between 18 and 25 kg/m^2^, and be able to understand and read the French language. Candidates were ineligible if they were smokers; were hypersensitive to prasugrel, ritonavir or constituents of the tablets; consumed alcohol regularly; had concomitant disease; or used any drug or food such as grapefruit in the month prior to the study that can either affect or be metabolized by CYP3A, 2C19, 2B6 and 2C9. Volunteers who used drugs associated with an increased risk of bleeding in the 10 days preceding the start of the study were excluded, as were those with a familial history of clotting disorders, antecedent of haemorrhagic disease, a previous or active gastrointestinal ulcer.

### Study design

This open-label cross-over study was conducted in 10 healthy volunteers at the clinical research unit of the Geneva University Hospitals. This study was conducted in accordance with Good Clinical Practice Guidelines and the Declaration of Helsinki. The Geneva University Hospital Ethics Committee and the Institutional Review Board Swissmedic approved the protocol before the study began. This clinical trial was registered at http://www.clinicaltrials.gov (NCT01346800). Written informed consent was obtained from each participant before the study. Volunteers received a single oral dose of 10 mg prasugrel on the first day. After at least one week, they received 100 mg ritonavir followed by 10 mg prasugrel 2 hr later. At each session, they also received a ‘micrococktail’ containing 10 mg bupropion, 5 mg flurbiprofen, 2 mg omeprazole and 0.1 mg midazolam for phenotyping of CYP2B6, CYP2C9, CYP2C19 and CYP3A, respectively, 1.5 hr after micrococktail administration. Adverse events were assessed during the study.

Each individual participated in the study for approximately 3 weeks, from initial screening to the final session.

### Analysis of DBS samples

Capillary blood samples for pharmacokinetic analysis were collected prior to and 0.25, 0.5, 1, 1.5, 2, 3, 4 and 6 hr after the administration of a single dose of prasugrel. After a small finger prick using disposable lancet (BD Microtainer: Contact-Activated Lancet, United Kingdom), 5 μl blood was spotted onto filter paper (Protein Saver 903 Card, Whatman) that had been previously soaked with 20 μl of 2-bromo-3’-methoxyacetophenone (BMAP) at 30 mM. This pre-treatment allows the unstable thiol group in prasugrel AM to form a disulphide bond with BMAP. Samples were stored at −20°C until analysis.

The DBS concentrations of prasugrel AM midazolam, omeprazole, bupropion and flurbiprofen were measured on a LC-MS/MS platform consisting of a 5500 QTtrap® triple quadrupole linear ion trap mass spectrometer equipped with a TurboIon Spray™ interface (AB Sciex, Darmstadt, Germany) and an Ultimate 3000 RS pump (Dionex) as the LC system. Data were acquired and processed using Analyst software (version 1.5.1; AB Sciex).

#### Analytical method description

Before analysis, discs (i.d. 6 mm) covering the entire DBS were punched out and placed in the bottom of individual LC vials containing a 300 μL inert insert (BGB Analytik, Germany). Samples were extracted by adding 100 μL of methanol containing 1 ng/ml derivatized prasugrel AM-d_3_ (Sirius Fine Chemicals, Germany). After 30 min., 5 μL of the supernatant was injected into the LC-MS/MS system.

The analytes were separated on a Kinetex Phenomenex (Brechbühler, Switzerland) RP C18 column (50 × 2.1 mm, 2.6 μm particles i.d.) using a gradient elution mode running from water–acetonitrile 98:2, v/v to water–acetonitrile 10:90, v/v for 2 min. The total run time was 6 min., including column wash and equilibration. After electrospray ionization (ESI), MS/MS detection was carried out with multiple reactions monitoring (MRM) at unit resolution operating in dual mode (positive and negative) with a time settling of 65 ms.

The instrument parameters were manually optimized as follows: curtain gas: 20 psi; ion source gas 1 and 2 (GS1 and GS2): 30 and 40 psi, respectively; ion source voltage: 5000 V in positive ESI mode and −4500 V in negative ESI mode; temperature: 650°C; entrance potential and collision cell exit potential: 10 V in positive ESI mode and −10 V in negative mode. The MRM transitions of the analytes relevant to this study are summarized in table 1.

**Table 1:** Multiple reactions monitoring parameters for the detection of prasugrel, prasugrel active metabolite, prasugrel active metabolite-d3, midazolam, omeprazole, bupropion and flurbiprofen

Compounds	Polarity	Q1→Q3 (m/z)	CE (V)	DP (V)	Dwell time (ms)	LLQ (ng/ml)
Prasugrel	+	374→206	23	100	2	0.5
Prasugrel AM^1^	+	498→348	28	100	2	0.5
Prasugrel AM-d_3_^1^	+	501→348	25	150	2	0.5
Midazolam	+	326→291	37	165	2	0.1
Omeprazole	+	346→198	19	66	2	0.5
Bupropion	+	240→131	30	80	2	0.5
Flurbiprofen	+	243→199	18	50	2	100

CE, collision energy; DP, declustering potential; LLQ, lower limit of quantification.

1 Compounds derivatized with 2-bromo-3-methoxyacetophenone (BMAP).

#### Validation

The method was fully validated according to the guidelines of the European Medicines Agency on validation of bioanalytical methods based on three non-consecutive days of testing 16. A typical validation day consisted of 5 DBS calibrators (Cal) injected in duplicate (n = 2) and 4 DBS quality controls (QC) injected in quadruplicate (n = 4). Cal and QC DBS samples were prepared independently in the same way using fresh human EDTA blood (supplied by the University Hospitals of Geneva). The calibration curves were linear over the standard concentration ranges of 0.1–50 ng/ml for midazolam; 0.5–250 ng/ml for prasugrel AM, omeprazole and bupropion; and 50–10,000 ng/ml for flurbiprofen. Based on these calibration curves, the trueness expressed as relative bias and the intermediate precision of the QCs was calculated. The values were within the expected criteria (±15%) for all the analytes. The selectivity of the method was investigated by injecting DBS pre-treated with BMAP obtained from the 10 volunteers recruited in the study. No interfering peaks were observed in the retention window of the analytes nor were there any matrix effects (<15%).

To ensure that the DBS specimens collected during the study could be shipped and stored at ambient temperature for a short period, the analytes’ stability was investigated over 30 days at three different temperatures (i.e. −20°C, +4°C and room temperature). The assay showed that all compounds were stable at the tested temperatures, as back-calculated concentrations were comprised between 85% and 115% of the corresponding 1-day-old DBS samples. As no significant difference was observed between storage at room temperature and at −20°C, DBS cards could be collected, shipped and stored at ambient temperature. Moreover, reinjection of study DBS samples that were stored at −20°C for 6 months showed similar results to those previously obtained.

### Phenotyping in plasma samples

A blood sample (approximately 6 ml) was obtained 1.5 hr after administration of the ‘micrococktail’ at each session for phenotyping. Blood samples were immediately centrifuged and the separated plasma was stored at −20°C until analysis. We used the validated extraction methods routinely used in our laboratory. Omeprazole, flurbiprofen, midazolam and their metabolites were extracted as described previously [17]. Bupropion was extracted by a liquid–liquid extraction procedure. All probes and their metabolites were analysed by the LC–MS/MS method described in the previous section.

### Size calculation

To detect an inhibition of prasugrel AM formation by ritonavir of at least 20 ± 10% with a power of 80% and α = 0.05, eight volunteers were needed. Two additional volunteers were recruited in case of volunteer withdrawal.

### Statistics

The pharmacokinetic parameters of prasugrel AM, midazolam, bupropion, flurbiprofen and omeprazole were calculated using a non-compartmental method by WinNonLin® version 5.2 (Pharsight, Mountain View, CA, USA). All statistical tests were performed with spss version 17 (Chicago, IL, USA). According to regulatory guidelines, results were presented as the mean values (±S.D.) or as mean ratios with 90% confidence intervals. The values for *t*_max_ were presented as median (range). The effect of ritonavir on the pharmacokinetic variables of prasugrel was estimated with the Wilcoxon signed rank test using spss version 17. All statistical tests were interpreted at the 5% significance level (two-sided).

## Results

### Volunteers

Ten healthy male volunteers were enrolled in the study. Volunteers were between 21 and 41 years of age with a mean (±S.D.) of 26 ± 6 years. Volunteers ranged in height from 165 to 185 cm, weighed between 63 and 95 kg and had a mean body mass index (±S.D.) of 22.8 ± 2 kg/m^2^. They were in good health, as determined by review of their medical history and physical examination. All 10 volunteers successfully completed the study. No adverse effects were reported during the study and all volunteers were included in the pharmacokinetic analysis. One volunteer was excluded from the evaluation of CYP2C19 activity because omeprazole and its metabolite were not detectable in his plasma and DBS.

### Prasugrel pharmacokinetics

**Fig. 2:**
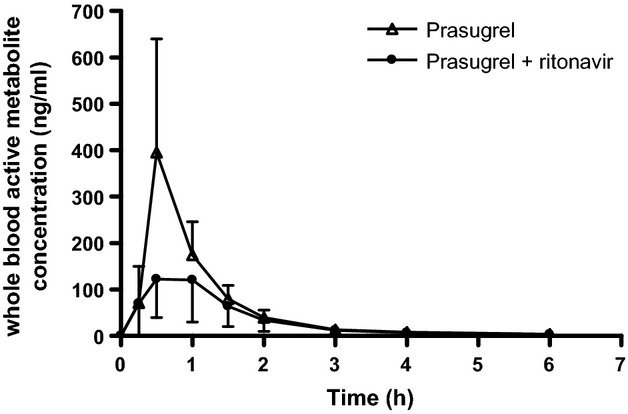
Mean (±S.D.) whole blood concentrations for prasugrel's active metabolite in dried blood spots after prasugrel alone (Δ) or prasugrel with ritonavir (•). Values are shown as the mean ± S.D.

**Fig. 3:**
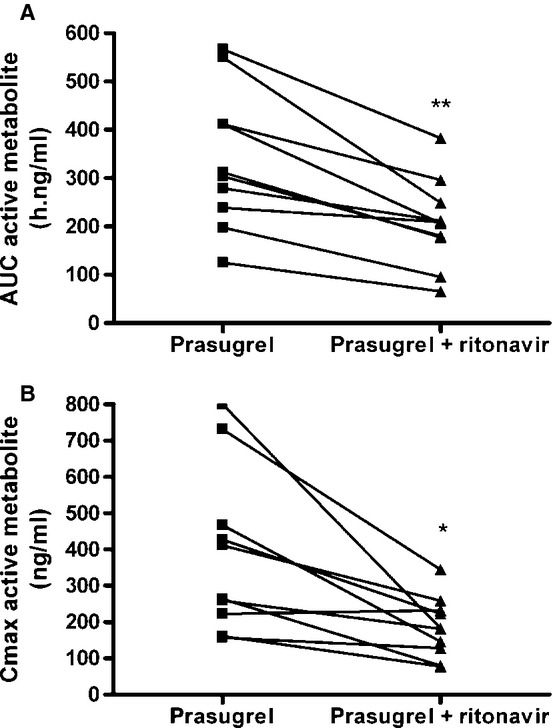
Effect of ritonavir on individual (A) AUC_0–6 hr_ or (B) *C*_max_ of the prasugrel active metabolite after administration of 10 mg prasugrel alone or 10 mg prasugrel with 100 mg ritonavir. *p < 0.05, **p ≤ 0.005.

In comparison with prasugrel alone, AUC_0–6 hr_ and *C*_max_ decreased by 38.4% (mean ratio: 0.62, CI 90%: 0.54; 0.7, *p* = 0.005) and 44.9% (mean ratio: 0.55, CI 90%: 0.4; 0.7, *p* = 0.007), respectively, when ritonavir was administered with prasugrel. However, ritonavir did not affect the time to reach maximum concentration (*t*_max_; median ratio: 1, CI 90%: 0.5; 2, *p* = 0.73) and half-life (*t*_1/2_; mean ratio: 0.94, CI 90%: 0.66; 1.22, *p* = 0.33) of prasugrel AM (figs 2 and 3; table 2).

**Table 2:** Pharmacokinetic parameters of prasugrel active metabolite

	Prasugrel alone	Prasugrel + ritonavir	Ratio (prasugrel + ritonavir *versus* prasugrel alone)	CI 90%	*p*-value
*t*_max_, hr	0.5 (0.5–1)	0.5 (0.25–1.5)	1	0.5–2	0.73
*t*_1/2_, hr	1.62 (0.54)	1.42 (0.66)	0.94	0.66–1.22	0.33
*C*_max_, ng/ml	389.8 (226.2)	185.4 (83.1)	0.55	0.40–0.7	**0.007**
AUC, hr ng/ml	339.6 (144.7)	207.5 (91.1)	0.62	0.54–0.7	**0.005**

Values are presented as the means (±S.D.) for *t*_1/2_, *C*_max_ and AUC and as median (range) for *t*_max_ values or mean ratios (prasugrel + ritonavir *versus* prasugrel alone) with 90% confidence intervals (CI 90%).

*t*_max_, time to reach maximal concentration; *t*_1/2_, elimination half-life; *C*_max_, maximal concentration; AUC, area under the concentration-time curve; Significant values (*p* < 0.05) were highlighted in bold.

### Cytochrome P450 activities

**Fig. 4:**
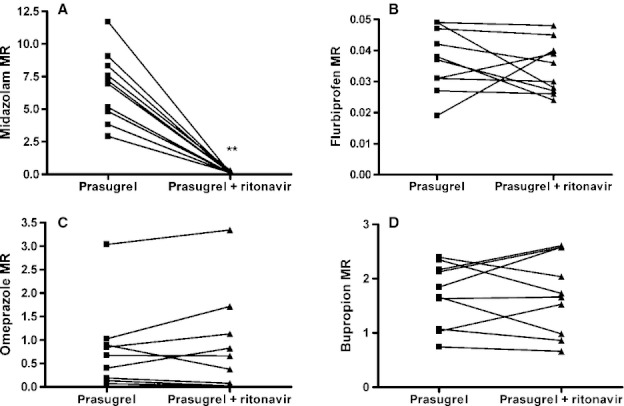
Effect of ritonavir on individual metabolic ratios of (A) midazolam, (B) flurbiprofen, (C) omeprazole and (D) bupropion after administration of 10 mg prasugrel or 10 mg prasugrel with 100 mg ritonavir. *p < 0.05, **p ≤ 0.005.

Midazolam MR was 97.8 ± 1.4% lower after ritonavir administration compared with the values for prasugrel alone (mean ratio: 0.022, CI 90%: 0.014; 0.03, *p* = 0.005). When ritonavir was co-administered with prasugrel, midazolam AUC_0–6 hr_ and *C*_max_ were 26 times and 6 times, respectively, the values obtained for prasugrel alone. Omeprazole MR (mean ratio: 0.92, CI 90%: 0.49; 1.35, *p* = 0.59), AUC_0–6 hr_ (mean ratio: 1.25, CI 90%: 0.94; 1.57, *p* = 0.44) and *C*_max_ (mean ratio: 1.54, CI 90%: 0.77; 2.32, *p* = 0.44) were not affected by ritonavir, and neither were flurbiprofen MR (mean ratio: 1.02, CI 90%: 0.78; 1.26, *p* = 0.58), AUC_0–6 hr_ (mean ratio: 0.95, CI 90%: 0.84; 1.05, *p* = 0.44) or *C*_max_ (mean ratio: 0.98, CI 90%: 0.82; 1.14, *p* = 0.72). Unexpectedly, bupropion MR (mean ratio: 1.02, CI 90%: 0.85; 1.19, *p* = 0.88), AUC_0–6 hr_ (mean ratio: 1, CI 90%: 0.93; 1.1, *p* = 0.96) and *C*_max_ (mean ratio: 1.03, CI 90%: 0.85; 1.21, *p* = 0.96) were not influenced by the co-administration of ritonavir and prasugrel (table 3; fig. 4).

**Table 3:** Pharmacokinetic parameters estimated from dried blood spots analysis and metabolic ratios of midazolam, bupropion, omeprazole and flurbiprofen measured in plasma

	Prasugrel alone	Prasugrel + ritonavir	Ratio	CI 90%	*p*-value
Midazolam					
*C*_max_	0.17 (0.09)	0.75 (0.29)	6.1	(3.5; 8.7)	**0.005**
AUC	0.31 (0.26)	2.53 (1.12)	26.5	(1.8; 51.3)	**0.005**
Metabolic Ratio (OH midazolam/midazolam)	6.74 (2.64)	0.13 (0.07)	0.022	(0.014; 0.03)	**0.005**
Bupropion					
*C*_max_	20.9 (6.3)	21.5 (8.2)	1.03	(0.85; 1.21)	0.96
AUC	49.1 (14.1)	49.3 (16.1)	1	(0.93; 1.1)	0.96
Metabolic Ratio (OH bupropion/bupropion)	1.70 (0.59)	1.72 (0.73)	1.02	(0.85; 1.19)	0.88
Omeprazole					
*C*_max_	35.6 (32.1)	37.3 (32.5)	1.54	(0.77; 2.32)	0.44
AUC	81.9 (96.5)	96.5 (111.9)	1.25	(0.94; 1.57)	0.44
Metabolic Ratio (OH omeprazole/omeprazole)	0.92 (1.13)	1.1 (1.25)	0.92	(0.49; 1.35)	0.59
Flurbiprofen					
*C*_max_	1135.7 (684.4)	1095 (653.8)	0.98	(0.82; 1.14)	0.72
AUC	3872.7 (2178.1)	3679.4 (2132.8)	0.95	(0.84; 1.05)	0.44
Metabolic Ratio (OH flurbiprofen/flurbiprofen)	0.037 (0.01)	0.034 (0.008)	1.02	(0.78; 1.26)	0.58

Values are presented as the means (±S.D.) or mean ratios (prasugrel + ritonavir *versus* prasugrel alone) with 90% confidence intervals (CI 90%).

*C*_max_, maximal concentration; AUC, area under the concentration-time curve; Significant values (*p* < 0.05) were highlighted in bold.

## Discussion

This study shows that prasugrel pharmacokinetics is significantly altered by ritonavir. In fact, ritonavir significantly decreased prasugrel AM AUC_0–6 hr_ and *C*_max_ while *t*_max_ and *t*_1/2_ were not affected.

As determined from a previous *in vitro* study, 38–70% of the bioactivation of oxo-prasugrel to prasugrel AM is performed by CYP3A4 and 2–26% is performed by CYP2B6 [2]. In the present study, ritonavir was used to investigate the effect of the inhibition of CYP3A and possibly other CYPs on the prasugrel AM pharmacokinetics.

In the present study, ritonavir totally inhibited CYP3A4/5 activity as indicated by the decrease of more than 97% of midazolam MR and the increase of midazolam's AUC_0–6 hr_ to a level 26 times that of observed without ritonavir, and the increase of midazolam's *C*_max_ to a level 6 times that observed without ritonavir. These results are in agreement with previous studies demonstrating that ritonavir is a very potent CYP3A inhibitor [11,18]. A previous study assessing the effect of ketoconazole on prasugrel pharmacokinetics and pharmacodynamics demonstrated a decrease in prasugrel AM *C*_max_ while AUC and platelet aggregation were not affected [5]. These contradictory results might be due to a higher CYP3A inhibition potential of ritonavir in comparison with ketoconazole [19]. In fact, ritonavir inhibited CYP3A more strongly than ketoconazole, as indicated by its increase of midazolam's AUC by a factor 28.4 while ketoconazole increased it by a factor 10–15 [18,20,21]. The pharmacokinetic differences between the results of ketoconazole and ritonavir could be explained by the mechanism of inhibition of the CYP3A4 by these molecules: while ritonavir is a competitive and non-competitive irreversible inhibitor, ketoconazole is only a competitive reversible inhibitor of CYP3A4.

Regarding CYP2B6 activity, ritonavir did not significantly affect either bupropion or 5-hydroxybupropion pharmacokinetics. This may be explained by the high Ki (5 μM) value of CYP2B6, which is higher than the expected ritonavir *C*_max_ after a 100 mg dose (1–2 μM) [22]. Higher concentrations of ritonavir might therefore be necessary to highlight any CYP2B6 inhibition measured through bupropion metabolism. Furthermore, omeprazole MR, flurbiprofen MR and their pharmacokinetic parameters did not change after ritonavir treatment.

One limitation of this study was that ritonavir was administered as a single dose. Ritonavir is one of the most potent known inhibitors of CYP3A but may have an induction effect when administered long-term [23]. However, this remains controversial, because some *in vivo* studies have failed to demonstrate induction and instead reported persistent CYP3A inhibition, even after long-term use [24]. The data obtained in our study are consistent with a pharmacokinetic drug–drug interaction between prasugrel and ritonavir *via* a potent inhibition of CYP3A4/5. Pharmacodynamic measurements were not performed in this study as prasugrel was administered in single dose. Nevertheless, according to some studies, from a clinical point of view, decreases in prasugrel AM production may be associated with lower platelet inhibition [25,26].

In conclusion, the inhibition of the bioactivation pathways of prasugrel by ritonavir mainly via CYP3A4/5 significantly affected prasugrel AM pharmacokinetics. This study highlights the importance of very carefully considering drugs affecting CYPs that might be prescribed concomitantly with prasugrel. Prasugrel is metabolized mainly by the CYP3A isoform that is inhibited, for example, by imatinib, ketoconazole and other antiretroviral drugs. CYP2B6, which is involved in the metabolism of prasugrel, also metabolizes other drugs such as methadone, cyclophosphamides and tramadol. Further PK/PD studies are needed to investigate the impact of these CYP-mediated drug–drug interactions on patients receiving prasugrel treatment and particularly in HIV patients with acute coronary syndrome simultaneously treated by prasugrel and ritonavir.

## References

[1] Wiviott SD, Antman EM, Winters KJ, Weerakkody G, Murphy SA, Behounek BD (2005). Randomized comparison of prasugrel (CS-747, LY640315), a novel thienopyridine P2Y12 antagonist, with clopidogrel in percutaneous coronary intervention: results of the Joint Utilization of Medications to Block Platelets Optimally (JUMBO)-TIMI 26 trial. Circulation.

[2] Rehmel JL, Eckstein JA, Farid NA, Heim JB, Kasper SC, Kurihara A (2006). Interactions of two major metabolites of prasugrel, a thienopyridine antiplatelet agent, with the cytochromes P450. Drug Metab Dispos.

[3] Thummel KE, Wilkinson GR (1998). In vitro and in vivo drug interactions involving human CYP3A. Annu Rev Pharmacol Toxicol.

[4] Turpeinen M, Raunio H, Pelkonen O (2006). The functional role of CYP2B6 in human drug metabolism: substrates and inhibitors in vitro, in vivo and in silico. Curr Drug Metab.

[5] Farid NA, Payne CD, Small DS, Winters KJ, Ernest CS, Brandt JT (2007). Cytochrome P450 3A inhibition by ketoconazole affects prasugrel and clopidogrel pharmacokinetics and pharmacodynamics differently. Clin Pharmacol Ther.

[6] Farid NA, Small DS, Payne CD, Jakubowski JA, Brandt JT, Li YG (2008). Effect of atorvastatin on the pharmacokinetics and pharmacodynamics of prasugrel and clopidogrel in healthy subjects. Pharmacotherapy.

[7] Farid NA, Jakubowski JA, Payne CD, Li YG, Jin Y, Ernest IC (2009). Effect of rifampin on the pharmacokinetics and pharmacodynamics of prasugrel in healthy male subjects. Curr Med Res Opin.

[8] Daali Y, Ancrenaz V, Bosilkovska M, Dayer P, Desmeules J (2011). Ritonavir inhibits the two main prasugrel bioactivation pathways in vitro: a potential drug–drug interaction in HIV patients. Metabolism.

[9] Knox TA, Oleson L, von Moltke LL, Kaufman RC, Wanke CA, Greenblatt DJ (2008). Ritonavir greatly impairs CYP3A activity in HIV infection with chronic viral hepatitis. J Acquir Immune Defic Syndr.

[10] Mathias AA, West S, Hui J, Kearney BP (2009). Dose–response of ritonavir on hepatic CYP3A activity and elvitegravir oral exposure. Clin Pharmacol Ther.

[11] Yeh RF, Gaver VE, Patterson KB, Rezk NL, Baxter-Meheux F, Blake MJ (2006). Lopinavir/ritonavir induces the hepatic activity of cytochrome P450 enzymes CYP2C9, CYP2C19, and CYP1A2 but inhibits the hepatic and intestinal activity of CYP3A as measured by a phenotyping drug cocktail in healthy volunteers. J Acquir Immune Defic Syndr.

[12] Hesse LM, von Moltke LL, Shader RI, Greenblatt DJ (2001). Ritonavir, efavirenz, and nelfinavir inhibit CYP2B6 activity in vitro: potential drug interactions with bupropion. Drug Metab Dispos.

[13] Barfield M, Spooner N, Lad R, Parry S, Fowles S (2008). Application of dried blood spots combined with HPLC-MS/MS for the quantification of acetaminophen in toxicokinetic studies. J Chromatogr B Analyt Technol Biomed Life Sci.

[14] Beaudette P, Bateman KP (2004). Discovery stage pharmacokinetics using dried blood spots. J Chromatogr B Analyt Technol Biomed Life Sci.

[15] Clark GT, Haynes JJ, Bayliss MA, Burrows L (2010). Utilization of DBS within drug discovery: development of a serial microsampling pharmacokinetic study in mice. Bioanalysis.

[16] EMA http://www.ema.europa.eu/docs/en_GB/document_library/Scientific_guideline/2011/08/WC500109686.pdf.

[17] Jerdi MC, Daali Y, Oestreicher MK, Cherkaoui S, Dayer P (2004). A simplified analytical method for a phenotyping cocktail of major CYP450 biotransformation routes. J Pharm Biomed Anal.

[18] Greenblatt DJ, Peters DE, Oleson LE, Harmatz JS, MacNab MW, Berkowitz N (2009). Inhibition of oral midazolam clearance by boosting doses of ritonavir, and by 4,4-dimethyl-benziso-(2H)-selenazine (ALT-2074), an experimental catalytic mimic of glutathione oxidase. Br J Clin Pharmacol.

[19] Eagling VA, Back DJ, Barry MG (1997). Differential inhibition of cytochrome P450 isoforms by the protease inhibitors, ritonavir, saquinavir and indinavir. Br J Clin Pharmacol.

[20] Chien JY, Lucksiri A, Ernest CS, Gorski JC, Wrighton SA, Hall SD (2006). Stochastic prediction of CYP3A-mediated inhibition of midazolam clearance by ketoconazole. Drug Metab Dispos.

[21] Olkkola KT, Backman JT, Neuvonen PJ (1994). Midazolam should be avoided in patients receiving the systemic antimycotics ketoconazole or itraconazole. Clin Pharmacol Ther.

[22] Sekar V, Spinosa-Guzman S, De Paepe E, Stevens T, Tomaka F, De Pauw M (2010). Pharmacokinetics of multiple-dose darunavir in combination with low-dose ritonavir in individuals with mild-to-moderate hepatic impairment. Clin Pharmacokinet.

[23] Dixit V, Hariparsad N, Li F, Desai P, Thummel KE, Unadkat JD (2007). Cytochrome P450 enzymes and transporters induced by anti-human immunodeficiency virus protease inhibitors in human hepatocytes: implications for predicting clinical drug interactions. Drug Metab Dispos.

[24] Wyen C, Fuhr U, Frank D, Aarnoutse RE, Klaassen T, Lazar A (2008). Effect of an antiretroviral regimen containing ritonavir boosted lopinavir on intestinal and hepatic CYP3A, CYP2D6 and P-glycoprotein in HIV-infected patients. Clin Pharmacol Ther.

[25] Brandt JT, Payne CD, Wiviott SD, Weerakkody G, Farid NA, Small DS (2007). A comparison of prasugrel and clopidogrel loading doses on platelet function: magnitude of platelet inhibition is related to active metabolite formation. Am Heart J.

[26] Payne CD, Li YG, Small DS, Ernest CS, Farid NA, Jakubowski JA (2007). Increased active metabolite formation explains the greater platelet inhibition with prasugrel compared to high-dose clopidogrel. J Cardiovasc Pharmacol.

